# HTsort: Enabling Fast and Accurate Spike Sorting on Multi-Electrode Arrays

**DOI:** 10.3389/fncom.2021.657151

**Published:** 2021-06-21

**Authors:** Keming Chen, Yangtao Jiang, Zhanxiong Wu, Nenggan Zheng, Haochuan Wang, Hui Hong

**Affiliations:** ^1^Key Laboratory of Radio Frequency Circuit and System, Hangzhou Dianzi University, Hangzhou, China; ^2^Qiushi Academy for Advanced Studies, Zhejiang University, Hangzhou, China

**Keywords:** spike sorting, multi-electrode arrays, overlapping spikes, clustering, template match, HDBSCAN

## Abstract

Spike sorting is used to classify the spikes (action potentials acquired by physiological electrodes), aiming to identify their respective firing units. Now it has been developed to classify the spikes recorded by multi-electrode arrays (MEAs), with the improvement of micro-electrode technology. However, how to improve classification accuracy and maintain low time complexity simultaneously becomes a difficulty. A fast and accurate spike sorting approach named HTsort is proposed for high-density multi-electrode arrays in this paper. Several improvements have been introduced to the traditional pipeline that is composed of threshold detection and clustering method. First, the divide-and-conquer method is employed to utilize electrode spatial information to achieve pre-clustering. Second, the clustering method HDBSCAN (hierarchical density-based spatial clustering of applications with noise) is used to classify spikes and detect overlapping events (multiple spikes firing simultaneously). Third, the template merging method is used to merge redundant exported templates according to the template similarity and the spatial distribution of electrodes. Finally, the template matching method is used to resolve overlapping events. Our approach is validated on simulation data constructed by ourselves and publicly available data and compared to other state-of-the-art spike sorters. We found that the proposed HTsort has a more favorable trade-off between accuracy and time consumption. Compared with MountainSort and SpykingCircus, the time consumption is reduced by at least 40% when the number of electrodes is 64 and below. Compared with HerdingSpikes, the classification accuracy can typically improve by more than 10%. Meanwhile, HTsort exhibits stronger robustness against background noise than other sorters. Our more sophisticated spike sorter would facilitate neurophysiologists to complete spike sorting more quickly and accurately.

## 1. Introduction

Most neurons communicate with each other through firing action potentials. Part of the work of neurophysiologists is to understand the working mechanisms of the nervous system by studying these action potentials. One way to obtain action potentials is to use physiological electrodes for *in-vivo* extracellular recordings (Perge et al., 2014; Rey et al., 2015; Wu et al., 2018). Depending on the purpose of the study, neurophysiologists may wish to sort these action potentials according to putative firing units (Lewicki, 1998; Hill et al., 2011; Quiroga, 2012; Harris et al., 2016; Lefebvre et al., 2016; Carlson and Carin, 2019). Spike sorting aims to classify the spikes (action potentials) emitted from different units based on some degree of reliability.

In the early stage of this field, spike sorting was employed to sort extracellular recordings that were monitored only by a single electrode (McNaughton et al., 1983; Gray et al., 1995; Lewicki, 1998; Harris et al., 2000; Csicsvari et al., 2003). During that period, many algorithms had been reported to classify spikes (Lewicki, 1998; Pillow et al., 2013; Ekanadham et al., 2014; Franke et al., 2015; Rey et al., 2015; Carlson and Carin, 2019). However, single-electrode mode limits the development of spike sorting and neural information decoding. With the development of integrated circuits and electrode technology in recent decades, multi-electrode arrays (MEAs) which integrate multiple electrodes at a high density with tens of microns of pitch have become increasingly popular to acquire the neural signals for neuro-physiological experiments (Einevoll et al., 2012; Lefebvre et al., 2016; Pachitariu et al., 2016; Steinmetz et al., 2018; Carlson and Carin, 2019). MEAs provide more valuable temporal and spatial information that will facilitate the development of spike sorting (Lewicki, 1998; Einevoll et al., 2012; Lopez et al., 2016; Carlson and Carin, 2019).

However, researchers need to face new challenges when MEAs offer them opportunities to classify spikes better. First, most traditional algorithms cannot be employed to multi-electrodes because their computational time presents exponential growth as the number of electrodes increases (Einevoll et al., 2012; Lefebvre et al., 2016; Rossant et al., 2016; Steinmetz et al., 2018; Carlson and Carin, 2019). Second, neural signals can be captured possibly by multiple adjacent electrodes under a dense arrangement, and these signals can no longer be considered as independent existences. Meanwhile, overlapping spikes can disrupt the feature space to cause a decrease in classification accuracy (Lewicki, 1998; Einevoll et al., 2012; Carlson and Carin, 2019). Third, the tricky problems that exist in the single-electrode case, such as burst-firing neurons and electrode drift, are still troublesome (Bar-Hillel et al., 2006; Chestek et al., 2007; Calabrese and Paninski, 2011; Pachitariu et al., 2016; Steinmetz et al., 2018). To solve the problem of spike sorting in the high-density multi-electrode condition, numerous researchers have proposed their solutions. The Kilosort algorithm defines a loss function for spike waveform features and obtains optimal results by optimizing the loss function. HerdingSpikes and MountainSort developed their clustering methods for spike sorting by combining waveform features and electrode spatial location, but neither of them proposed the solutions for addressing overlapping events (Chung et al., 2017; Hilgen et al., 2017). YASS (Lee et al., 2017) proposed a different strategy of triage-then-cluster-then-pursuit. The strategy first excludes overlapping spikes, then clusters the remaining spikes. The experimental results demonstrated that this strategy is effective. However, a neural network in YASS is used to detect spikes, and the neural network needs a large number of labeled samples for training in the first place, which leads to YASS not being available in unsupervised applications. YASS new version (2.0) can train its neural network on new data, making it also unsupervised. However, training the neural network for the first run can be time consuming. Besides, a clustering method based on Gaussian hybrid models is adopted, but the distribution of samples is not always ideal Gaussian-shape, which means that the density-based or hierarchical clustering is a preferred choice (Lewicki, 1998; Rey et al., 2015). SpykingCircus (Yger et al., 2018) runs the clustering method to only obtain the templates. Then, the classification of the whole spikes is completed through the template matching method. In this way, it gets more accurate results, but relatively consumes more time on the template matching method.

In this paper, a fast and accurate multi-electrode spike sorting approach named HTsort is proposed. An important divide-and-conquer method is used to process data on multiple electrodes. Next, the clustering algorithm HDBSCAN (hierarchical density-based spatial clustering of applications with noise) is applied to classify clusters and detect overlapping events. Then, an automated template merging method is designed to address overclustering issue. Last, the template matching method is adopted to resolve the overlapping events. This paper is organized in the following ways. Section 2 elucidates the methodological details. Section 3 presents the performance test results. The discussion and conclusion is placed in section 4.

## 2. Materials and Methods

### 2.1. Overview

**Figure 1:**
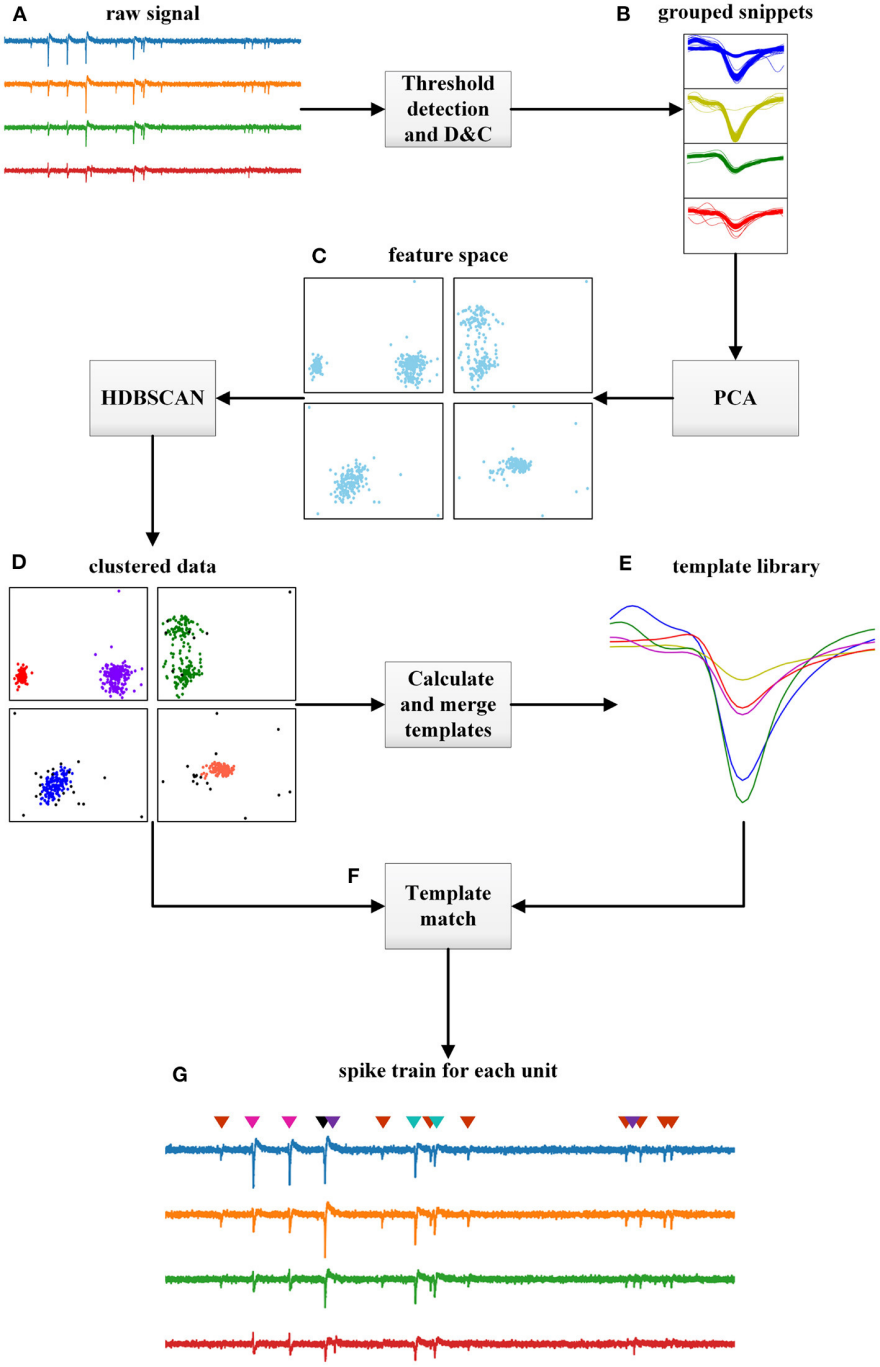
Flowchart of the proposed HTsort, illustrated with a 4-electrode dataset. **(A)** Raw signals acquired by multi-electrodes. **(B)** Snippets extracted by threshold detection and grouped with the divide-and-conquer (D&C) method. **(C)** Feature space in each group generated with PCA. **(D)** Clusters and outliers found by HDBSCAN in each group. **(E)** The template library is constructed after finishing the template merging. **(F)** The overlapping spikes resolved by the template matching method. **(G)** Spike sorting results.

The proposed approach can be illustrated by Figure 1. First, the neural signals are acquired by multiple electrodes and then the threshold detection method is used to detect spikes. Before this, some pre-processing can be included depending on the situation, such as using a bandpass filter with a bandwidth of 300-3kHz. The threshold detection step performs amplitude detection parallelized on each channel to pick up signal fragments (snippets) that contain the full spike waveforms.

After the threshold detection, the divide-and-conquer method groups snippets by electrodes. Specifically, if some snippets are acquired on electrode *ch*_*A*_, then they are grouped into group *gp*_*A*_. This grouping actually utilizes the electrode location feature to pre-cluster the spikes based on the assumption that different units have different locations (Einevoll et al., 2012; Lefebvre et al., 2016; Rossant et al., 2016; Hilgen et al., 2017). Without the help of pre-cluster, spikes with very similar waveforms emitted by different units would be hard to separate by only a clustering method based on waveform features. Following this, principal component analysis (PCA) is performed to carry out feature extraction and data decomposition for each snippet vector, and eventually the feature space for each snippet group is‘constructed.

Next, HDBSCAN is carried out in each snippet group to cluster samples (McInnes et al., 2017). For high-density multi-electrode cases, it is not surprising that several spike waveforms overlap, and such colliding events can often become outliers in the feature space (Lewicki, 1998; Franke et al., 2009; Ekanadham et al., 2014; Lefebvre et al., 2016; Lee et al., 2017; Yger et al., 2018). Different from some conventional clustering method can be disturbed by these outliers, HDBSCAN can mark them as noise and perform hierarchical density-based spatial clustering on the remaining samples. In this way, the classification of benign spikes is finished. And the possible decrease of classification accuracy caused by overlapping events can be avoided.

After the clustering method, the cluster centers that estimated by calculating the average of samples belong to the same cluster are used to construct the template library. If the number of templates obtained is more than the ground-truth, overclustering (overfitting of clusters) has occurred. The reason is that snippets that should belong to the same putative neuron are divided into different groups by the divide-and-conquer method (Pachitariu et al., 2016; Lee et al., 2017; Yger et al., 2018; Carlson and Carin, 2019). The automated template merging method is used to address this problem based on two criteria: the Euclidean distance between their centers is close enough, and the spatial position of the electrodes is near enough. The first criterion ensures that the two templates are close enough in PCA space, which means they have similar waveform features. The second criterion ensures that the spatial locations where they appear are sufficiently close, and therefore likely to be emitted by the same neuron. By this, a more compact and precise template library is obtained and the overclustering problem is solved. By the way, we do not store and maintain a template library for long. A template library is only used temporarily during once spike sorting. Therefore, we build a standalone template library during each spike sorting instead of taking a permanent public template library.

Finally, the overlapping events that were previously temporarily excluded by HDBSCAN are addressed by the template matching. The template matching method subtracts the best matching template from the overlapping event iteratively until there is no more valid spike component. In this way, the overlapping events get resolved.

### 2.2. Pre-processing

Pre-processing of the raw signal is not necessary, but can usually help the following steps work better. The filtering removes some of the noise from the original signal and benefits the operation of the threshold detection. A bandpass filter with a bandwidth of 300–3 kHz is used in this step of HTsort. Then the data is interpolated or downsampled as appropriate. If the sampling rate of the original signal is low and without interpolation, the peak alignment and feature extraction will be affected (Rey et al., 2015). If the sampling rate is pretty high and without downsampling, excessive data points need to be checked by the threshold detection. In the experiment, we downsampled the original signals (30 kHz) to 14 kHz. More details about the influence of the sampling rate is discussed in section 4.2.

### 2.3. Threshold Detection

The threshold detection method is used to extract snippets that contain the full spike waveforms from the raw voltage signals. The principle behind it is that the most important feature that distinguishes spike signals from background noise is their larger amplitude (Lewicki, 1998). Therefore, it is sensible to set a threshold to distinguish them, but threshold setting requires some experience. If the threshold is too high, it leads to an increase in false-negative samples (missed spikes). If the threshold is too low, it leads to an increase in false-positive samples (the number of background events that cross the threshold) (Lewicki, 1998; Quiroga et al., 2004; Rey et al., 2015). An automated method for threshold determination based on an estimate of the standard deviation of background noise σ_*n*_ was proposed (Quiroga et al., 2004). The threshold can be set according to the following equation:

(1) $$\begin{array}{r} threshold=k*{\hat{\sigma }}_{n} \end{array}$$

(2) $$\begin{array}{r} {\hat{\sigma }}_{n}=\frac{\text{median }\left(\left|X\right|\right)}{0.6745} \end{array}$$

where *k* is typically a constant between 3 and 5, and *X* is the band-pass filtered signal. The denominator 0.6745 comes from the inverse of the cumulative distribution function for the standard normal distribution evaluated at 0.75. Generally, the majority part of extracellular recordings are background noise, so it is feasible to estimate σ_*n*_ with *X*. Additionally, adopting the median of |*X*| reduces the amplitude interference from spiking activity. In fact, this method of threshold setting has been validated on simulated (Quiroga et al., 2004) and real data (Quiroga, 2012) to provide a robust estimation of σ_*n*_, even if the noise distribution might deviate from Gaussian.

In the proposed HTsort, the alignment of the peaks of the waveforms is finished during the threshold detection. First, an appropriate window size *wnd* is determined to represent the length of the extracted waveform (e.g. 3 ms time). Next, define the position *p* for the peak alignment (e.g., the middle of the window). Then, the sliding window algorithm is applied to the band-pass filtered signal. When the absolute value of data point at position *p* is the maximum within the window and greater than *threshold*, then this snippet of length *wnd* is extracted. The threshold detection processes over multiple electrode channels are performed in parallel and then snippets that contain the spike waveforms corresponding to each channel are output.

### 2.4. Divide-and-Conquer for Multi-Electrode

The divide-and-conquer method aims to use the spatial location information of the electrodes to perform a pre-clustering, and then discards the redundant data from multiple electrodes. When doing the pre-clustering, we do not explicitly enter the coordinates of each electrode, but directly group snippets by different electrodes. For example, if some snippets are acquired on electrode *ch*_*A*_, then they are grouped into group *gp*_*A*_. Generally, it is difficult for clustering methods based on waveform features to classify spikes that have similar waveforms and belong to different neurons, but this way of pre-clustering can utilize spatial information to separate them. The pre-clustering is based on the assumption that different neurons have different spatial locations (Lefebvre et al., 2016). Also, the pre-clustering may accidentally separate spikes that should belong to the same class, so the template merging method described in section 2.6 is employed to solve the problem. Another issue is that when multiple electrodes capture the same spike will lead to redundant data (Lefebvre et al., 2016; Lee et al., 2017; Yger et al., 2018). It is unnecessary to keep copies of the same spike within multiple groups. What the divide-and-conquer method do is to select the best channel for each spike event. For the snippets belonging to the same spike event, only the snippet with the largest magnitude is kept. This process can be represented as,

(3) $$\begin{array}{r} \hat{W}i=\underset{amplitude}{\max}\left\{Wi,j|j\in Gi\right\} \end{array}$$

where Ŵ*i* is the best estimation of the spike waveform for spike *Si*, and *Gi* denotes the set of adjacent electrodes. *Wi, j* is the waveform of *Si* that is acquired at the *j*th electrode. Besides, after completing the divide-and-conquer method, groups can carry out their respective clustering tasks in parallel, which is obviously efficient than performing clustering on all samples together.

### 2.5. Feature Extraction and HDBSCAN Clustering

Before clustering, the principal component analysis (PCA) is used to extract features from spike waveforms to construct the feature space. By PCA, an ordered set of orthogonal basis vectors (principal components) that capture the largest variation can be found (Mishra et al., 2017). In fact, studies have shown that the first three components account for about 76% of the spike data variation (Lewicki, 1998; Rey et al., 2015; Lefebvre et al., 2016; Carlson and Carin, 2019). Typically, only the first two have variances that are significantly above the background noise, so using more components would offer little improvement in classification accuracy (Lewicki, 1998). Besides, using more components as the dimensionality of the clustering space may cause the “curse of dimensionality” in machine learning. Thus, only the first two components were selected in our experiments.

Then, the hierarchical density-based spatial clustering method HDBSCAN is used to accomplish the clustering task and outlier detection. HDBSCAN is an improvement from DBSCAN and it works by the following process: (1) Transform the feature space according to the definition of mutual reachability distance: *d*_mreach−*k*_(*a, b*) = max{core_*k*_(*a*), core_*k*_(*b*), *d*(*a, b*)}, where *d*(*a, b*) is the original metric distance between a and b, and *core*_*k*_(*x*) is the distance between x and its kth nearest neighbor. (2) Build a minimum spanning tree based on the mutual distances of samples in the new space. (3) Construct a cluster hierarchy from the minimum spanning tree. (4) Condense the cluster hierarchy and then extract the stable clusters (Daszykowski and Walczak, 2009; Campello et al., 2015; McInnes and Healy, 2017; McInnes et al., 2017). More details can be found on HDBSCAN documentation. In essence, HDBSCAN iteratively conducts DBSCAN with different parameters to yield more stable clustering results (McInnes et al., 2017). In this way, HDBSCAN can work well with variable sample density distributions, but DBSCAN does not (McInnes and Healy, 2017; McInnes et al., 2017).

The clustering method HDBSCAN is used in the proposed HTsort approach for several reasons: (1) If spike variability depends only on additive and Gaussian stationary background noise, all clustering methods based on Gaussian mixed models will be effective, but the reality is that non-Gaussian distribution is common in neural signals. So, using density-based or hierarchical clustering methods is preferable (Lewicki, 1998; Carlson et al., 2014; Rey et al., 2015; Lee et al., 2017). (2) Overlapping events usually occur as outliers in the feature space, which disrupt the proper functioning of the clustering method. HDBSCAN can perform outlier detection and exclude them.

**Figure 2:**
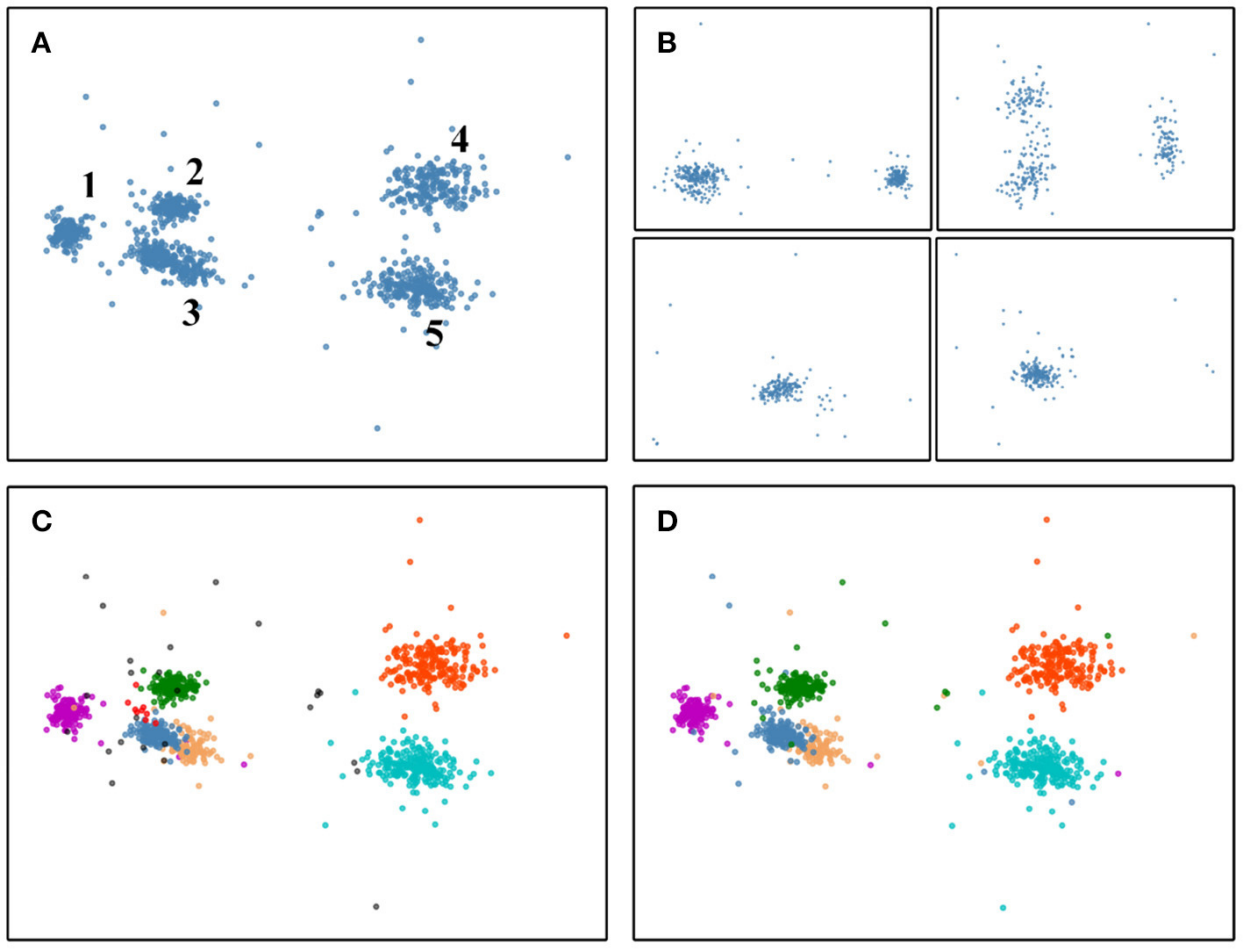
The pre-clustering effect of the divide-and-conquer can separate samples that are difficult to classify based on waveform features only. **(A)** Distribution of the overall samples in PCA space when not using the divide-and-conquer. But here we already removed the redundant data between the electrodes for display convenience. If look at intuitively, it contains five clusters. **(B)** Distribution in PCA space of the samples within each group resulting from the divide-and-conquer method. **(C)** Clustering is performed for each group in **(B)**, and the results are presented in the same PCA space. There are seven clusters and some black outliers. Outliers can be overlapping events and will be finally resolved by the template matching method. **(D)** The ground-truth of **(A)**, it contains six clusters. The steel-blue cluster and the sandy-brown cluster can not be split without using the divide-and-conquer to achieve pre-clustering. Although redundant red cluster is derived in **(C)**, this overclustering problem can be addressed by the template merging method.

The process from the divide-and-conquer method to HDBSCAN can be illustrated in Figure 2, the involved data is a four-electrode dataset (publicly available on SpikeForest website). The sample distribution of snippets output by the threshold detection method in the PCA space is shown in Figure 2A. Note that the PCA method is used in Figure 2A just for display convenience. In fact, the PCA method is only executed after the divide-and-conquer method is finished in Figure 2B. Also, we have removed the redundant data between the electrodes for display convenience. If look at intuitively, it contains five clusters in Figure 2A. Figure 2B shows the distribution of the samples in PCA space within each group after using the divide-and-conquer method. Since there are four electrodes, four groups are here. After the four groups have completed the HDBSCAN clustering in parallel, the results are presented in Figure 2C. Here are seven clusters and some outliers (outliers can be overlapping events and will be finally addressed by the template matching). Figure 2D indicates the ground-truth of the clusters, contrary to our intuition in Figure 2A, the number 3 cluster should be split into two classes. But after using the divide-and-conquer method, the steel-blue cluster and the sandy-brown cluster can be successfully separated in Figure 2C, which is the effect of pre-clustering (these two clusters are overlapping in the PCA space, and it is almost impossible to separate them by clustering algorithm based on waveform features). Although redundant red cluster is derived in Figure 2C, this overclustering problem can be addressed by the template merging method (see section 2.6).

### 2.6. Template Merging

After finishing clustering, the templates would be obtained by calculating the mean or median of the samples contained in each cluster. And each template can be used to represent a class of spike signals.

**Figure 3:**
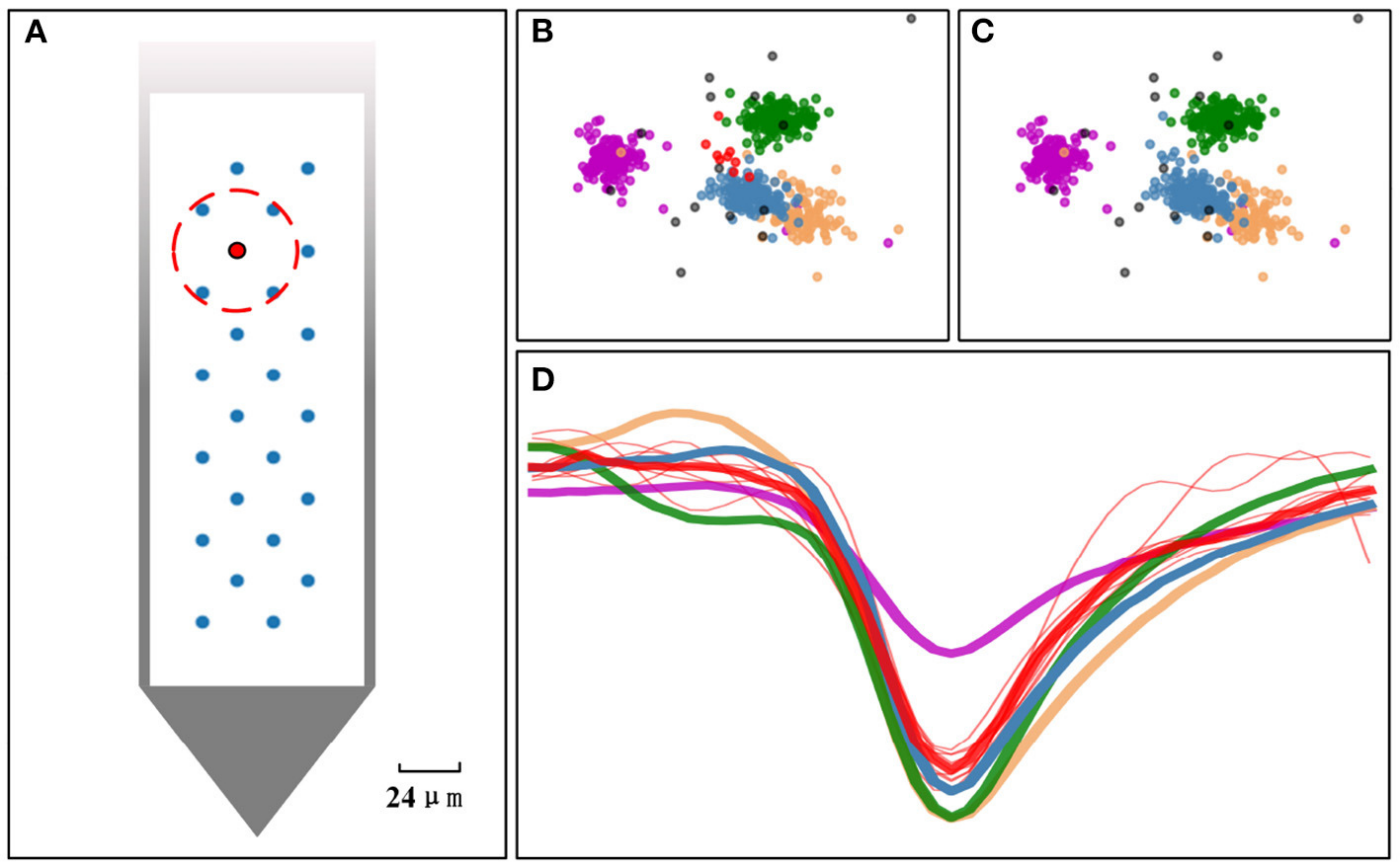
Overclustering issue addressed by the template merging. **(A)** The arrangement of the electrodes on Neuropixels-24 probe. If the blue electrodes within the red circle are identified as neighbors of the red electrode, the templates obtained on the red electrode have a chance to merge with the templates estimated from its neighbor. **(B)** Five clusters found in PCA space before doing the template merging. **(C)** The small red cluster is merged into the steel-blue cluster after performing the template merging. **(D)** Templates estimated from each cluster (thick line) and samples from red cluster (red thin line). If the similarity of two templates is greater than a set threshold and the electrodes they belong to are a pair of neighbor electrodes, the clusters they represent will be merged.

An action potential would often acquired by multiple electrodes. Although the best channel is selected for each spike signal by the divide-and-conquer method, the reality is that the best channel is not always the same one. If the neuron that emits spike *S* is as close to both electrodes *ch*_1_ and *ch*_2_, the best channel will sometimes be electrode *ch*_1_ and sometimes electrode *ch*_2_, resulting in the same template obtained in the group corresponding to both electrodes. If templates that belong to the same class of spikes are not merged, it will cause overclustering (overfitting of clusters) (Pachitariu et al., 2016; Lee et al., 2017; Yger et al., 2018; Carlson and Carin, 2019). The merging of templates follows two criteria: 1. The Euclidean distance between the two templates must be less than a specified tunable parameter σ (default setting is 0.05). 2. The electrodes that each of the two templates belonging to must be neighbors. The first criterion directly reflects how similar the two templates are. The second criterion is used to ensure that the two templates do need to be merged. After the template merging, a more compact and precise template library is gotten. The process of the template merging can be expressed as,

(4) $$\begin{array}{r} T_{merged}=\left(\frac{1}{m}\overset{m}{\underset{i=1}{\sum }}t_{i,1},\frac{1}{m}\overset{m}{\underset{i=1}{\sum }}t_{i,2},\ldots ,\frac{1}{m}\overset{m}{\underset{i=1}{\sum }}t_{i,n}\right) \end{array}$$

where *T*_*merged*_ is the final template vector got after merging *m* templates that satisfy the two criteria. Each template is represented by an n-dimensional PC feature vector. *t*_*i, j*_ is the *j*th feature of the ith template that need to be merged. As illustrated in Figure 3A, if the electrodes within the red circle are identified as neighbors of the red electrode, then the templates obtained on the red electrode have a chance to merge with the templates obtained on these neighbor electrodes. In our practical experiments, the distance-based threshold *thres*_*nb*_ is used to determine the neighbors of an electrode. If the distance between two electrodes is less than *thres*_*nb*_, then they are neighbors to each other. The setting of the threshold theoretically depends on the transmission distance of the action potential and the electrical properties of the channels. Since our merging criterion also needs to ensure that the templates are indeed similar enough, the threshold will not be as sensitive. So a choice of 20–50μm is reasonable.

The effect of performing the template merging on a 4-electrode dataset can be elucidated in Figures 3B,C. The case before performing the merging is showed in Figure 3B, and the result after performing the merging is showed in Figure 3C. The red cluster merged with the steel-blue cluster, because their centers (templates) in Figure 3D are close (similar) enough and their corresponding two electrodes are neighbors. However, the steel-blue cluster will not be merged with the sandy-brown cluster, nor will the red cluster be merged with the green cluster, as they cannot satisfy both criteria for merging.

### 2.7. Template Matching

Template matching can find the areas in a target that best match a template based on specified metrics. One use of template matching in spike sorting is served as an alternative to clustering methods to accomplish classification, as is the case in Kilosort and SpykingCircus (Pachitariu et al., 2016; Yger et al., 2018). But in general, clustering methods are better able to handle uncertainty in template shape and spike assignments than template matching (Lee et al., 2017). Another use of template matching is employed to resolve overlapping events. Although it is possible to use outlier detection to identify overlapping spikes and to keep them out of downstream processing, we would like to recover their labels as many as possible (Lewicki, 1998).

In the proposed HTsort, the template matching method takes the following steps to address overlapping events: (1) Find a template from the template library that matches best the overlapping event. (2) Define a criterion to accept the template. (3) If the template is accepted, subtract it from the overlapping event and go back to the first step. This method is referred to as greedy templates matching pursuit (Rey et al., 2015; Lefebvre et al., 2016; Pachitariu et al., 2016). In the proposed HTsort, the templates needed for the matching process come from the template library after performing the template merging method, and the metric used for matching can be represented by,

(5) $$\begin{array}{r} R\left(x,y\right)=\frac{\underset{x^{\prime },y^{\prime }}{\sum }{\left(T\left(x^{\prime },y^{\prime }\right)-I\left(x+x^{\prime },y+y^{\prime }\right)\right)}^{2}}{\sqrt{\underset{x^{\prime },y^{\prime }}{\sum }T{\left(x^{\prime },y^{\prime }\right)}^{2}\cdot \underset{x^{\prime },y^{\prime }}{\sum }I{\left(x+x^{\prime },y+y^{\prime }\right)}^{2}}} \end{array}$$

where *I* is the overlapping event and *T* is a template from the template library. (*x, y*) is the starting point of the target signal to be matched and (*x*′, *y*′) is the offset during the matching process. In step (2) we accept the template that yields the maximum *R*(*x, y*) > 0.8. After subtracting the template from raw data in step (3), if the amplitude of the residuals is less than the parameter *dtcTh* (its value is set equal to the parameter *threshold* of the threshold detection step), it is assumed that there is no longer a valid spike signal component and the whole process stops.

## 3. Results

### 3.1. Data

One hurdle for the current development of spike sorting is the lack of standardized measures and validation data. Many automated spike sorting algorithms already exist, but usually the accuracy is validated by different paper authors under their own experimental conditions. Potential biases may exist (Einevoll et al., 2012; Rey et al., 2015; McInnes and Healy, 2017; Buccino and Einevoll, 2020; Magland et al., 2020; Wouters et al., 2020). To address this problem, a platform is needed to provide open, uniform, and standard spike sorting datasets. With such a platform, numerous spike sorting algorithms can be validated and compared in a fair and reasonable manner. SpikeForest is a reproducible, continuously updating platform which benchmarks the performance of spike sorting codes across a large curated database of electrophysiological recordings with ground truth (Magland et al., 2020). In this paper, both simulation data generated by MEArec and publicly available data from SpikeForest are used to validated the proposed HTsort approach (datasets are available at Github and SpikeForest website). Then the proposed HTsort was compared with several state-of-the-art spike sorting algorithms on the same datasets by using the unified framework SpikeInterface. SpikeInterface wraps many popular spike sorters and provides interfaces for comparison and benchmarking (Buccino et al., 2020). In addition, we prepared the simulation datasets in a control variables way, to show the classification accuracy and execution time of the algorithm when the number of electrodes increases and when the number of neural units increases, respectively.

The specifications of the simulation datasets generated by MEArec are reported in Table 1. First, the number of neurons is fixed to 10 and the number of electrodes is gradually increased from 4 to 128. Second, the number of electrodes is fixed to 32 and the number of neurons gradually increased from 4 to 15. So, there are 9 different dataset specifications. And ten recordings for each specification are prepared with different random seeds (affects the random selection of spike templates and the random generation of background noise). Except for tetrode recordings, the probe types of remaining recordings are Neuropixels. Neuropixels probes are nearly the most popular in the electrophysiology field, and they provide dense recording sites on a narrow shank, with on-board amplification, digitization, and multiplexing (Steinmetz et al., 2018). The details of the used publicly available dataset from SpikeForest are reported in Table 2.

**Table 1:** Simulation data prepared for performance comparison.

**Dataset**	**Num**.	**Sample**	**Duration**	**Num**.	**Num**.	**Num**.	**Probe type**
	**Recordings**	**rate(Hz)**	**(s)**	**Electrodes**	**Units**	**Spikes**	
C4-U10	10	30,000	30	4	10	7,438	tetrode
C24-U10	10	30,000	30	24	10	7,438	Neuropixels-24
C32-U10	10	30,000	30	32	10	7,438	Neuropixels-32
C64-U10	10	30,000	30	64	10	7,438	Neuropixels-64
C128-U10	10	30,000	30	128	10	7,438	Neuropixels-128
C32-U4	10	30,000	30	32	4	3,225	Neuropixels-32
C32-U6	10	30,000	30	32	6	4,582	Neuropixels-32
C32-U8	10	30,000	30	32	8	6,044	Neuropixels-32
C32-U10	10	30,000	30	32	10	7,438	Neuropixels-32
C32-U15	10	30,000	30	32	15	11,356	Neuropixels-32

**Table 2:** “HYBRID_JANELIA” dataset publicly available on the SpikeForest website.

**Serial**	**Dataset**	**Num**.	**Sample**	**Duration**	**Num**.	**Num**.	**Num**.
**number**		**recordings**	**rate(Hz)**	**(s)**	**electrodes**	**Units**	**Spikes**
0	4c_600 s	3	30,000	600	4	74	241,585
1	16c_600 s	3	30,000	600	16	74	241,585
2	32c_600 s	3	30,000	600	32	74	241,585
3	64c_600 s	2	30,000	600	64	74	241,585
4	4c_1,200 s	3	30,000	1,200	4	74	482,868
5	16c_1,200 s	3	30,000	1,200	16	74	482,868
6	32c_1,200 s	3	30,000	1,200	32	74	482,868
7	64c_1,200 s	1	30,000	1,200	64	74	482,868

### 3.2. Experimental Results

**Figure 4:**
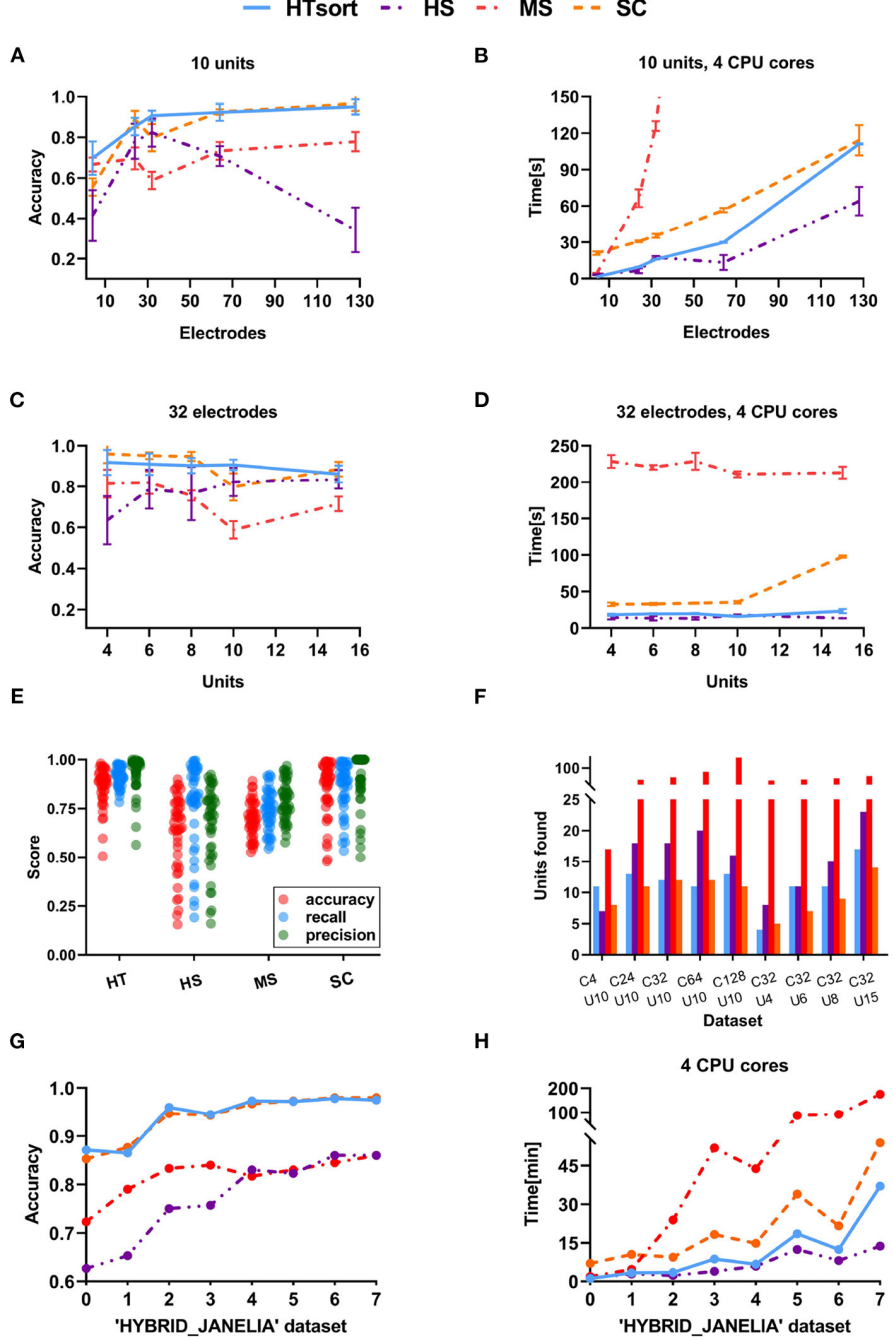
Performance comparison between the art-of-the-state sorters. **(A)** Accuracy of the sorters evaluated on simulation data with the various number of electrodes. **(B)** Time consumption of the sorters evaluated on simulation data with a various number of electrodes. **(C)** Accuracy of the sorters evaluated on simulation data with the various number of neurons. **(D)** Time consumption of the sorters evaluated on simulation data with a various number of neurons. The proposed HTsort achieves the best trade-off between accuracy and time consumption. **(E)** The accuracy, recall, precision of sorters evaluated on simulation data. **(F)** The number of units detected by sorters on simulation data. **(G,H)** Accuracy and time consumption of the sorters evaluated on “HYBRID_JANELIA” dataset.

The proposed HTsort has been validated on simulation data reported in Table 1 and compared with HerdingSpikes (HS, Hilgen et al., 2017), MountainSort (MS, Chung et al. 2017), and SpykingCircus (SC, Yger et al. 2018) in x86_64 Ubuntu 18.04 environment. The sorters mentioned above all work in the unsupervised situation. Other existing sorters that cannot work in unsupervised scenarios (e.g. YASS) are not involved in the comparison. The detailed results are reported in Figures 4A–F and Tables 3, 4. The definitions of metrics involved in the results are:

(6) $$\begin{array}{r} accuracy=\frac{tp}{tp+fp+fn} \end{array}$$

(7) $$\begin{array}{r} precision=\frac{tp}{tp+fp} \end{array}$$

(8) $$\begin{array}{r} recall=\frac{tp}{tp+fn} \end{array}$$

where tp is the number of true positive events (correct classification), and fp is the number of false positive event and fn is the number of false negative event (misclassification).

**Table 3:** Classification accuracy of HTsort, HerdingSpikes, MountainSort, and SpykingCircus tested on simulation data.

**Dataset**	**Num**.	**Num**.	**Num**.	**HTsort**	**Herding**	**Mountain**	**Spyking**
	**recordings**	**electrodes**	**Units**	**(%)**	**spikes (%)**	**sort (%)**	**circus (%)**
C4-U10	10	4	10	69.7(±8.2)	41.4(±12.6)	66.6(±3.5)	55.5(±4.3)
C24-U10	10	24	10	85.3(±4.3)	78.0(±8.6)	69.7(±5.4)	88.9(±4.1)
C32-U10	10	32	10	90.6(±2.5)	82.3(±7.0)	58.8(±4.3)	79.8(±6.7)
C64-U10	10	64	10	92.3(±4.1)	70.8(±4.9)	73.4(±4.4)	92.5(±1.3)
C128-U10	10	128	10	95.0(±3.8)	34.2(±11.1)	77.9(±4.7)	96.6(±3.6)
C32-U4	10	32	4	91.9(±6.1)	63.6(±11.8)	81.4(±6.9)	95.9(±4.4)
C32-U6	10	32	6	91.0(±5.3)	78.7(±9.5)	81.9(±5.6)	95.1(±1.6)
C32-U8	10	32	8	90.3(±3.8)	76.6(±13.1)	75.5(±2.5)	94.8(±2.2)
C32-U10	10	32	10	90.6(±2.5)	82.3(±7.0)	58.8(±4.3)	79.8(±6.7)
C32-U15	10	32	15	86.2(±4.2)	83.5(±4.6)	71.5(±3.6)	88.6(±3.6)
Collision^*^	100	4120	930	46.3(±11.6)	34.7(±12.7)	29.9(±8.2)	49.2(±12.4)
Average	100	4120	930	87.8(±4.3)	69.7(±8.5)	70.5(±4.3)	85.8(±3.9)

* *Classification accuracy for collision/overlapping spikes*.

**Table 4:** Time consumption of HTsort, HerdingSpikes, MountainSort, and SpykingCircus tested on simulation data.

**Dataset**	**Num**.	**Num**.	**Num**.	**HTsort**	**Herding**	**Mountain**	**Spyking**
	**Recordings**	**electrodes**	**Units**	**(s)**	**spikes (s)**	**sort (s)**	**circus (s)**
C4-U10	10	4	10	1.3(±0.1)	3.3(±0.4)	3.8(±0.8)	21.1(±1.5)
C24-U10	10	24	10	9.6(±0.2)	6.5(±2.1)	66.5(±7.4)	30.8(±0.8)
C32-U10	10	32	10	15.9(±0.2)	17.7(±1.0)	125.9(±4.1)	35.4(±1.6)
C64-U10	10	64	10	29.9(±0.4)	13.4(±6.1)	574.4(±45.2)	56.5(±1.8)
C128-U10	10	128	10	111.1(±0.5)	64.0(±11.7)	2831.3(±21.6)	114.1(±12.5)
C32-U4	10	32	4	18.2(±1.5)	14.4(±2.7)	228.6(±9.0)	32.6(±2.5)
C32-U6	10	32	6	19.3(±1.0)	13.5(±3.2)	220.4(±3.2)	33.0(±1.5)
C32-U8	10	32	8	19.6(±1.1)	13.2(±1.7)	228.7(±11.8)	34.1(±0.7)
C32-U10	10	32	10	15.9(±0.2)	17.7(±1.0)	210.9(±4.1)	35.4(±1.6)
C32-U15	10	32	15	23.3(±3.0)	13.4(±0.4)	213.0(±8.2)	97.7(±1.4)

For an ideal multi-electrode spike sorting algorithm, when the number of neurons is fixed increasing the number of electrodes can help the algorithm to better resolve and classify spikes. Meanwhile, we want to maintain an acceptable linear relationship between the time consumption and the number of electrodes. As shown in Figure 4A, HTsort outperforms other methods in accuracy, showing a steady improvement in accuracy with an increasing number of electrodes. The results of SC are actually not bad, except for the poor performance when the number of electrodes is low (less than 64 electrodes). The performance of MS is mediocre. For HS, as the number of electrodes is gradually increased from 4 to 32, its classification accuracy clearly benefits from the increase of the number of electrodes. But as the number of electrodes continues to be increased the classification accuracy of HS declines rapidly. So in our test, it is shown that HS is unable to process datasets acquired from more than 64 electrodes properly. From Figure 4B, we found that the computation time of HTsort, HS, and SC algorithms have an approximate linear relationship with the number of electrodes. HS consumed least time overall, but its accuracy is not satisfactory. Then we compared the time consumption of HTsort and SC and it is clear that HTsort performs better. In the most disparate case (4-electrode), HTsort consumes <10% of the time of SC. Overall, HTsort has higher efficiency than SC. The time consumption changing curve of MS is unacceptable, and it clearly does not work well with the growing number of electrodes.

If the number of electrodes is fixed, an increase in the number of neurons means more complex classification. This will inevitably lead to a decrease in the accuracy of the algorithm, as well as an increase in time consumption. It is challenging that spike sorting methods maintain relatively high accuracy and relatively low time consumption. As shown in Figure 4C, HTsort has maintained high accuracy (over 90%). When the number of neurons is small (8 or less), the accuracy of SC can be about 5% higher than HTsort. But as the number of neurons increases, its accuracy fluctuates considerably, and in the worst case, the accuracy is 10% lower than HTsort. The performance of MS is still mediocre. As for HS, we can see that its accuracy is very unstable, and it is clear that HS is not robust enough to cope with the variations of the noise and spike waveforms. The time consumption was reported in Figure 4D. HS is still the fastest, and HTsort is close to HS. They both maintain their time consumption without an obvious increase. SC consumes more time than HTsort and HS. As for MS, its time consumption is many orders of magnitude higher than other algorithms.

The publicly available data reported in Table 2 are alse used to validate the above algorithms, and the results are reported in Figures 4G,H. Again, the proposed HTsort is able to provide high classification accuracy and low time consumption, which means that the proposed HTsort approach offers a good trade-off between accuracy and time consumption.

## 4. Discussion and conclusion

In comparison with other state-of-the-art spike sorts, it is validated that the proposed HTsort approach achieves a better trade-off between accuracy and time consumption. First, the divide-and-conquer method utilizes the spatial information of electrodes to achieve pre-clustering. And large computational tasks are divided into multiple small task groups that can be executed in parallel. Then, HDBSCAN is used to execute efficient clustering and outlier detection. Outlier detection is important because the outliers can disturb the clustering process. A subsequent template merging step is employed to rectify the clustering results. The final template merging step ensures the accurate parsing of overlapping events. Together, the HTsort outperforms other sorts.

SC is good enough, and its accuracy is close to HTsort (sometimes up to 5% more accurate than HTsort). But SC typically consumes 45% more time than HTsort. The reason is that SC only uses the clustering method to provide templates without exploiting the classification results from clustering method. And SC finally uses template matching to finish classification for all samples with spending more time for post-processing on each electrode (Lee et al., 2017; Yger et al., 2018). MS is moderately accurate but has more time consumption. One reason for this is that the clustering process in MS is repeatedly executed until no more clusters can be found to split. It is a good way to separate clusters that would be easily merged, but undoubtedly increase the time consumption (Chung et al., 2017). HS runs fast because it only needs to perform a mean shift clustering based on waveform features and spatial position. But its classification accuracy is unstable. One reason for this is that the mean shift clustering needs to assume that samples belonging to the same class are satisfying a spherical distribution in the feature space (Hilgen et al., 2017). The other reason is that HS is sensitive to noise and spatial position bias, which leads to its obvious fluctuating results on datasets with the same specification but variable background noise.

### 4.1. Classification Accuracy

In our experiments, the standard definition formula for classification accuracy in this field is used,

(9) $$\begin{array}{r} accuracy=\frac{tp}{tp+fp+fn} \end{array}$$

where *tp* is the number of true positive events (correct classification), and *fp* is the number of false positive event and *fn* is the number of false negative event (misclassification). For the threshold detection, as we mentioned in section 2.3, the setting of the threshold affects both *fp* and *fn*. But in the proposed HTsort, we can tend to set the threshold value relatively lower without significantly affecting the final classification accuracy. A low threshold setting leads to an increase in *fp*, which means that some background noise will be considered as spike signals. These background noise, although recognized in the threshold detection, are detected as outliers in HDBSCAN clustering because their waveform features present stochasticity and deviation. As we described in section 2.7, the possible spike components in the outliers are detected by the template matching method. If the outliers are overlapping events, the valid spike components would be detected. Whereas, background noise is discarded. This fact allows the threshold setting to be more lenient and enhances the robustness of HTsort. Classification accuracy of sorters that do not support outlier detection and analysis, will be more sensitive to the threshold setting and non-stationary background noise.

In the threshold detection method, we use the sliding window algorithm to complete the peak alignment of spikes. The peak alignment determines whether the features of extracted snippets can be aligned at the correct position, thus peak alignment can be an important factor for accurate classification of both template and feature-based algorithms (Lewicki, 1998). The sampling frequency greatly influences the effect of peak alignment. If the sampling frequency is high enough, then sufficient waveform features can be preserved and alignment can be done more easily. However, processing signals with a high sampling frequency can cause the whole algorithm to become computationally expensive, so a reasonable trade-off needs to be made. We have discussed in detail the effect of sampling frequency on time consumption in the below section 4.2.

For the divide-and-conquer method, it achieves pre-clustering by using the spatial location information of the electrodes, but without explicitly entering the coordinates of each electrode. The most important benefit of pre-clustering is that it can help classify spikes that have similar waveforms but belong to different neurons, which are often difficult to do with clustering based on waveform features. In the case of MEAs, the spatial information of multiple electrodes should be properly utilized to help improve the classification accuracy, and this form of pre-clustering is an example. However, we have to point out that in the case of severe electrode drift, the divide-and-conquer provides a limited pre-clustering effect. During several hours of electrode recording, relative movement between an electrode and biological tissue usually occurs (Lewicki, 1998; Rey et al., 2015). In such a case, spikes that are excited by different neurons and have similar waveforms can no longer be distinguished only by the electrode location information. To handling the drift and other non-stationarity, SpikingCircus and Kilosort are reported can offer good results because they do not rely on clustering to get the final results, and the clustering methods based on waveform features are thought to be more sensitive to waveform changes so clustering methods are not good at dealing with drift problem. One of our current attempts is to model the movement of the electrodes to be able to compensate for electrode position offsets. Further improvement of the divide-and-conquer method to cope with more extreme cases is one of our future research directions.

For the proposed HTsort, it is critical to coordinate the operations of HDBSCAN, the template merging, and the template matching. HDBSCAN performs clustering and outlier detection in the waveform feature space (outliers may be background noise or overlapping events). The superfluous templates resulting from overclustering are merged by the template merging according to the criteria detailed in section 2.6. The template matching uses templates to seek possible spikes in the outliers and to deconvolute the overlapping events. The combined performance of HDBSCAN and the template matching demonstrates the feasibility and efficiency of the triage-then-cluster-then-pursuit strategy proposed by YASS (Lee et al., 2017). However, the implementation of outlier detection in YASS relies on the training of a neural network. The role of this neural network is to extract spike waveforms from the original signal and screen overlapping events. Further, the clustering method DP-GMM employed by YASS (Lee et al., 2017) requires the assumption that the distribution of samples belonging to the same neuron obeys a multivariate Gaussian distribution. But many studies have proven that this is only the ideal case (Lewicki, 1998; Lee et al., 2017; McInnes et al., 2017; Yger et al., 2018). Here, we employed HDBSCAN to apply the strategy proposed by YASS into the unsupervised scenario, without the assumption of a distribution prototype. HDBSCAN executes density-based spatial clustering and supports outlier detection. Notably, there is no concern that *tp* samples may be identified as outliers, since they will be recovered in the template matching step. The template merging can merge the redundant clusters and provide a more accurate template library for the template matching. It also corrects the clustering results to a certain extent, enabling more robustness in the final classification results.

### 4.2. Time Consumption

After our experiments and analysis, the main time-consuming parts in the proposed HTsort are the threshold detection and HDBSCAN clustering. For the threshold detection, it is executed in parallel on the raw data of multiple electrodes. If the number of CPU cores of the computer is comparable to the number of electrodes, the time complexity of this process can be considered as *O*(*T* * *SPF*). Where T represents the duration of recording and SPF represents the sampling frequency. However, usually the number of CPU cores will be significantly less than the number of electrodes, then the time complexity becomes *O*(*T* * *SPF* * *C*), where C denotes the number of electrodes. In this case, to minimize the time consumption, we can choose to downsample the raw signals in the preprocessing step. Unfortunately, this may result in the loss of the spike waveform features to various degrees. The study of Navajas et al. (2014) discussed in detail that the minimum requirement for the sampling frequency without obviously affecting the classification accuracy is 7 kHz, while 100% fidelity can be achieved with 28 kHz. Considering the possible research limitations and timeliness of the study, we downsampled the original signals (30 kHz) to a conservative 14 kHz in our preprocessing step. In the future, we will comprehensively investigate the appropriate downsampling range to reasonably and effectively reduce the time consumption of the threshold detection.

For the time consumption of HDBSCAN, its authors have done an exhaustive benchmarking performance test on HDBSCAN documentation. The results show that the asymptotic complexity of HDBSCAN is sub-*O*(*n*^2^), but does not reach *O*(*n log*(*n*)), where *n* is the number of samples involved in clustering. In HTsort, as described in section 2.4 we use the divide-and-conquer method to remove redundant data (only one copy of each spike is retained) and execute HDBSCAN in parallel for each group. In this way, the number of samples involved in clustering is greatly reduced, and the time consumption can be further decreased by a parallel mechanism when the number of CPU cores is sufficient.

The template matching method is quite time-consuming, which slides a template over the target signal to find the best matching region. If a total of *N* templates and *M* target signals need to be matched, then the time consumption is proportional to *N* * *M*. In HTsort, we only use the template matching for overlapping events, and they are usually no more than 10% of the total samples (Lewicki, 1998; Quiroga, 2012; Rey et al., 2015; Carlson and Carin, 2019). If the template matching method is used to classify all samples (e.g. SpykingCircus), it will take much more time. The situation gets worse when the number of neurons increases. The time consumption of SpykingCircus (Yger et al., 2018) is rapidly increasing in Figure 4D for this reason.

## Data Availability Statement

The original contributions presented in the study are included in the article/supplementary material, further inquiries can be directed to the corresponding author/s.

## Author Contributions

KC: conceptualization, methodology, and writing-original draft. YJ: software, investigation, and writing-original draft. HH: project administration, writing-review, and editing. ZW: project administration, writing-review, and editing. NZ and HW: writing-review and editing. All authors contributed to the article and approved the submitted version.

## Conflict of Interest

The authors declare that the research was conducted in the absence of any commercial or financial relationships that could be construed as a potential conflict of interest.
